# The Likelihood of Extinction of Iconic and Dominant Herbivores and Detritivores of Coral Reefs: The Parrotfishes and Surgeonfishes

**DOI:** 10.1371/journal.pone.0039825

**Published:** 2012-07-11

**Authors:** Mia T. Comeros-Raynal, John Howard Choat, Beth A. Polidoro, Kendall D. Clements, Rene Abesamis, Matthew T. Craig, Muhammad Erdi Lazuardi, Jennifer McIlwain, Andreas Muljadi, Robert F. Myers, Cleto L. Nañola, Shinta Pardede, Luiz A. Rocha, Barry Russell, Jonnell C. Sanciangco, Brian Stockwell, Heather Harwell, Kent E. Carpenter

**Affiliations:** 1 IUCN Species Programme/SSC Marine Biodiversity Unit-Global Marine Species Assessment, Biological Sciences, Old Dominion University, Norfolk, Virginia, United States of America; 2 School of Marine Biology and Aquaculture, James Cook University, Townsville, Queensland, Australia; 3 School of Biological Sciences, The University of Auckland, Auckland, New Zealand; 4 Silliman University, Dumaguete, Negros Oriental, Philippines; 5 Department of Marine Sciences and Environmental Studies, University of San Diego, San Diego, California, United States of America; 6 Conservation International, Denpasar, Bali, Indonesia; 7 Department of Environment and Agriculture, Curtin University, Perth, Western Australia, Australia; 8 Coral Triangle Center, Sanur, Bali, Indonesia; 9 Coral Graphics, Wellington, Florida, United States of America; 10 College of Science and Mathematics, University of the Philippines Mindanao, Davao City, Philippines; 11 Wildlife Conservation Society, Indonesia Marine Program, Bogor, Jawa Barat, Indonesia; 12 California Academy of Sciences, San Francisco, California, United States of America; 13 School of Environmental and Life Sciences, Charles Darwin University, c/- Arafura Timor Research Facility, Brinkin, Northern Territory, Australia; 14 Biological Sciences, Old Dominion University, Norfolk, Virginia, United States of America; Leibniz Center for Tropical Marine Ecology, Germany

## Abstract

Parrotfishes and surgeonfishes perform important functional roles in the dynamics of coral reef systems. This is a consequence of their varied feeding behaviors ranging from targeted consumption of living plant material (primarily surgeonfishes) to feeding on detrital aggregates that are either scraped from the reef surface or excavated from the deeper reef substratum (primarily parrotfishes). Increased fishing pressure and widespread habitat destruction have led to population declines for several species of these two groups. Species-specific data on global distribution, population status, life history characteristics, and major threats were compiled for each of the 179 known species of parrotfishes and surgeonfishes to determine the likelihood of extinction of each species under the Categories and Criteria of the IUCN Red List of Threatened Species. Due in part to the extensive distributions of most species and the life history traits exhibited in these two families, only three (1.7%) of the species are listed at an elevated risk of global extinction. The majority of the parrotfishes and surgeonfishes (86%) are listed as Least Concern, 10% are listed as Data Deficient and 1% are listed as Near Threatened. The risk of localized extinction, however, is higher in some areas, particularly in the Coral Triangle region. The relatively low proportion of species globally listed in threatened Categories is highly encouraging, and some conservation successes are attributed to concentrated conservation efforts. However, with the growing realization of man's profound impact on the planet, conservation actions such as improved marine reserve networks, more stringent fishing regulations, and continued monitoring of the population status at the species and community levels are imperative for the prevention of species loss in these groups of important and iconic coral reef fishes.

## Introduction

Parrotfishes (Labridae, Scarinae) and surgeonfishes (Acanthuridae) are among the most conspicuous and dominant groups of fishes on coral reefs, both in terms of numbers of individuals and biomass [1], [2]. Many species have wide distributional ranges, strong associations with coral reef environments [1], and achieve their highest species diversity in the Indo-Australian region [2], [3], particularly in the Indo-Malay-Philippine Archipelago or Coral Triangle region [4]–[6]. The parrotfishes are comprised of 10 genera and 100 valid species [7], [8]. Recent phylogenetic studies [9], [10] conclude that the Scaridae and the Odacidae should be subsumed in the family Labridae. We recognize the taxonomic disagreements in the sub-order Labroidei [7] but follow the placement of the scarines and odacines under Labridae as suggested by Westneat and Alfaro (2005) and Clements et al. (2004). The surgeonfishes are comprised of six genera and 82 valid species [2], [11], [12].

Species in these two families have long been considered to play important functional roles in coral reef ecosystems. They exhibit a range of feeding modes and ingest a variety of food items including plant material, detritus/bacterial complexes, zooplankton, live coral, and sessile/benthic invertebrates [13], [14]. Parrotfishes play key functional roles as grazers and bioeroders in the reef ecosystem [15]–[17], and are important components of the herbivore/detritivore functional feeding groups of tropical and sub-tropical reefs [18]–[23]. Parrotfishes primarily feed on detrital aggregates that are either scraped from the reef surface (epilithic) or excavated from the deeper reef substratum (endolithic) [24], while surgeonfishes demonstrate a diversity of feeding habits, more so than other groups of herbivorous fishes [2], with most members consuming living plant material or detrital aggregates [24], [25].

Parrotfishes and surgeonfishes have been collectively categorized as herbivores [14], [26]–[30], implying that they feed directly on living plant material [31]. The trophic biology in these groups is, however, complex with a variety of dietary items ingested other than living plant material. This study follows the approach to herbivory used by recent studies on the nutritional ecology of parrotfishes and surgeonfishes [24], [25], [32]–[36], many of which have defined herbivory by post-ingestive processes rather than focusing on feeding behavior and the structure and functionality of the jaws. Due to trophic diversification in these groups, various species will have different impacts on the sessile biota of reefs and the patterns of energy flow and nutrient and material cycling within reef ecosystems.

Herbivorous fishes are considered to play an important role in coral reef dynamics by limiting the establishment and growth of algal communities that impede coral recruitment [37]–[39] and providing the link for the flow of energy to higher trophic levels [13]. They also have the potential to influence the distribution and composition of algal assemblages in coral reef systems and to influence rates of production and internal composition [13]. Moreover, territoriality among herbivorous fishes can shape benthic communities and increase within-territory coral diversity through protection against predators [40].

The larger taxa of the excavating parrotfishes may especially have profound effects on the dynamics of reef growth and sedimentation [16], [41]. The Bumphead Parrotfish (*Bolbometopon muricatum*), the largest of all parrotfishes and the largest coral predator, is considered an important bioeroder [42]. Each individual has the capacity to remove five tonnes of carbonate annually from the reef, half of which is living coral [16]. For smaller-bodied excavators like the Daisy Parrotfish (*Chlorurus sordidus*), there is a non-linear relationship between body size and ecological function, such that reefs impacted by fishing and dominated by small individuals may be functionally impaired [43]. Indeed, the large excavating members of the parrotfish fauna are considered to perform an important functional role (e.g. facilitating bioerosion and aid in coral recruitment by prevention of algal overgrowth ) on present day coral reefs [16], [44]–[46].

Many species of parrotfishes and surgeonfishes are prized components of coral reef fisheries in many parts of their wide distributional range, with a number of dedicated fisheries particularly in the Caribbean [17], [47]–[49] and Western and Central Pacific [50]–[56]. Coral reef fisheries typically occur in developing countries and involve multi-species fisheries with varying degrees of target preference, gear usage, and habitat utilization [57]. In the last 10–20 years, increased fishing pressure has led to population declines in *B. muricatum* [52], [53], Rainbow Parrotfish (*Scarus guacamaia*) [58], and localized losses in several species of parrotfish and surgeonfish in the Philippines [59]–[61]. The threat of overfishing is compounded with the heightened risks that coral reef ecosystems face due to a number of anthropogenic pressures [62]–[65] and climate change events [66]–[69]. In particular, the decline of reef habitats is worrying for species that recruit into live coral [70]–[72], especially as recent studies show that habitat-specialists are those most vulnerable to extinction [64], [67], [73]. Habitat degradation coupled with fisheries exploitation can negatively impact the populations of these coral reef fishes [69], [74], [75] and could potentially lead to devastating consequences for the human communities that depend on the parrotfishes and surgeonfishes for food.

As iconic reef inhabitants, parrotfishes and surgeonfishes are also important components of marine park tourism and the diving industry. In addition, many are popular marine aquarium species, some of which are collected by the tens of thousands each year, such as the Yellow Tang (*Zebrasoma flavescens*) [76]. This species is the most collected aquarium fish in Hawai'i [76], [77] and accounts for 80% of the fish caught for the aquarium trade in the western coast of the Big Island in Hawai'i in recent years [76], [78]. While there have been concerns of declining populations in parts of this species' range, *Z. flavescens* is well monitored and is subject to a number of management actions including: marine reserves, Fish Replenishment Areas (FRAs), and establishment of a limited entry program for the aquarium fishery [79], [80].

Parrotfishes and surgeonfishes are ecologically and economically important, yet very little is known on the global impact of coral reef habitat loss on these reef-dependent species or the specific effects of systematic fishing on species and important functional groups. Information generated from comprehensive collection of species-specific assessments can not only identify the presence of threatened populations, but is important for refining conservation priorities, including designation and delineation of critical habitat, no-take zones or marine protected areas, or to inform policies that regulate resource extraction. For these reasons, species-specific data for each of the world's 179 known species of parrotfish and surgeonfish were collated to 1) determine the global conservation status of these fishes, 2) draw attention to regional importance and the local threats affecting species in these two families, 3) highlight the habitat status of coral reef dependent species, and 4) underline life history traits that affect coral reef dynamics and predispose species to heightened risks of extinction.

## Materials and Methods

### Red List Process

Global Red List Assessments were conducted for the world's known parrotfishes and surgeonfishes using the International Union for Conservation of Nature (IUCN) Red List Categories and Criteria [81]. A total of 179 species were assessed at two workshops held in Bali, Indonesia in 2009 and Cebu, Philippines in 2010. The three recently described species of parrotfishes and surgeonfish (*Acanthurus tractus*, *Sparisoma rocha*, and *Sparisoma choati*) are currently under assessment and are not included in these results. Eastern Tropical Pacific endemics were assessed in 2008 [82], and nine species were assessed using the sampled approach to the Red List Index in 2009. The sample Red List Index methodology was developed to facilitate application of the Red List to a wider scope of taxonomic groups, thereby providing a better representation of the status of the world's biodiversity [83]. Regional assessments using the Guidelines for Application of IUCN Red List Criteria at Regional Levels [84] were conducted for 18 commercial parrotfishes and surgeonfishes in the Indo-Malay-Philippines Archipelago (Coral Triangle region).

Prior to the workshops, species-specific information on taxonomy, distribution, population status and trends, ecology, biology, life history, utilization, impacts of major threats, and conservation measures were compiled. During the Red List Assessment workshops, species were evaluated one at a time by regional and international experts, with outside consultation and follow-up conducted when additional information was needed but not available at the workshop. Based on the most current data, each species was assigned to one of eight levels of extinction risk expressed as an IUCN Red List Category [85]. The Red List assessment process consolidates the most current and highest quality data available and ensures peer-reviewed scientific consensus on the likelihood of extinction for each species [85]–[88]. All species accounts and results of the Red List assessments are publicly accessible online on the IUCN Red List of Threatened Species website (http://www.iucnredlist.org).

There are eight different levels of extinction risk on the IUCN Red List Categories: Extinct (EX), Extinct in the Wild (EW), Critically Endangered (CR), Endangered (EN), Vulnerable (VU), Near Threatened (NT), Least Concern (LC), and Data Deficient (DD). A species is listed in one of the three threatened Categories (CR, EN, or VU) if it meets the thresholds and conditions for that category in one of the five different available criteria (A–E) (Table 1). A category of Near Threatened is assigned to a species that comes close to, but does not fully meet, all the thresholds or conditions required for a threatened category under one of the five extinction risk Criteria. Listing under category of Least Concern is assigned when there are no known threats to a species, or quantification of known threats for a species does not come close to meeting any of the threatened category thresholds. The Data Deficient category is applied when there is insufficient information available to adequately apply the criteria, such as taxonomic uncertainty, lack of key biological information, or inability to adequately quantify the impact of known threats.

**Table 1:** Summary of IUCN criteria for listing in a threat category (Vulnerable, Endangered, or Critically Endangered) (Sadovy et al. 2012).

**Criteria A**	**Population Reduction**: Size of population has been observed, estimated, or inferred to have declined by a considerable proportion (minimum 30%) over the past three generations.
**Criteria B**	**Geographic Range**: Species has a small range (maximum 20,000 km∧2) **and** is either (a) severely fragemented, (b) experiencing decline in range area or number of mature individuals, or (c) is experiencing extreme fluctuations in range area or number of mature individuals.
**Criteria C**	**Small Population Size and Decline:** Number of mature individuals is small (maximum 10,000) **and** there is continuing decline (minimum 10%) expected over the next three generations **or** (a) a continuing decline in the number or percent of mature individuals in each subpopulation, (b) extreme fluctuations in the number of mature individuals.
**Criteria D**	**Very Small or Restricted Population:** Number of mature individuals is less than 1000 **and/or** the area of occupancy is less than 20 km∧2 **or** ≤5 locations.
**Criteria E**	**Quantitative Analysis**: Quantitative population analysis indicates the probability of extinction in the wild to be ≥10% in the next 100 years.

### Application of Criteria to Parrotfishes and Surgeonfishes

The five Criteria underscore the real strength of the IUCN Red List as these quantitative thresholds are based on extinction risk theory and can be collectively applied across a range of taxonomic groups that exhibit diverse life histories [85], [89], [90]. Four of the five species of parrotfishes and surgeonfishes that qualified for a threatened or Near Threatened Category were assessed under Criterion A, which is based on quantifying population reduction over the greater time period of three generation lengths or 10 years. Population declines for these species were calculated using catch landings statistics and fishing effort information taken from local sources. Fishery-independent data collected using underwater visual censuses and informal knowledge (i.e., interviews with fishers) were employed when fishery dependent data was unavailable either regionally or by country. Life history information such as age at first maturity and longevity were used to calculate generation length, a measure of reproductive turnover to calculate population declines over a species-specific time period [81]. One species qualified for a threatened Category under Criterion D, which is used for species with a very small or restricted population. There were no parrotfish or surgeonfish species that met the thresholds and conditions for a threatened Category under the remaining Criteria B, C, or E (see Table 1 for description of Categories).

### Spatial Analyses

Digital distribution maps were created in ArcView 3.3 based on drawing a minimum convex polygon connecting points of known species' occurrence. Since the majority of parrotfishes and surgeonfishes inhabit shallow waters in coral reef habitats, each species map was cut to a maximum depth of 200 m with a 100 km buffer from the coastline, based on 2 minute spatial bathymetry data available from NOAA National Marine Fisheries Service (ETOPO1). This “cookie-cutter” method allows the analyses to be standardized and is a better representation of the known occurrence of these groups that inhabit shallow waters [91]. For analyses of biodiversity patterns, species' polygons were stacked and converted to a 10 km by 10 km raster grid using a geoprocessing script (cf. [5]). This script assigns a value for each cell that corresponds to the number of overlapping species distributions at the cell location, thus representing species richness per cell.

To determine the number of species affected by fisheries, distribution maps for all species that were impacted by the fisheries threat (targeted fisheries, including commercial, artisanal, and recreation catch and by-catch), were overlain to create a richness map for this type of threat. The two other major threat codes that were assigned, if relevant, to species during the workshop process included habitat loss (including that from coastal development) and pollution (including climate change).

The proportion of marine protected area within each species range was calculated in ArcGIS by first determining the total area of each species distribution map as described above. Each species distribution map was then overlain with the 2010 World Database of Protected Areas [92] to calculate the proportion of each species range that is within a Marine Protected Area.

### Designation of Habitat Type and Feeding Group

During Red List assessment workshops, participants assigned each species to one or more of the IUCN marine habitat classifications [93]. These pre-determined habitat classifications were developed for documenting taxa on the IUCN Red List in order to ensure standardization when describing the major habitat/s that a species occupies. Based on these classifications, the majority of the parrotfishes and surgeonfishes inhabit the marine neritic zone which comprises 10 habitat types: pelagic, subtidal rock and rocky reefs, subtidal loose rock/pebble/gravel, subtidal sandy, subtidal sandy/mud, subtidal muddy, macroalgal/kelp, coral reef, seagrass, and estuaries. Coral reef habitat is further subdivided into 6 categories: outer reef channel, back slope, foreslope, lagoon, inter-reef soft substrate and inter-reef rubble substrate. If applicable, some species were also classified under one or more of the four habitat types found in the inter-tidal and supra-tidal zones: sea cliffs and rocky offshore islands, mangrove submerged roots, rocky shorelines, and tidepools. To calculate the percentage of habitat utilization across all species, total species presence in each of the 14 habitat types was summed and divided by the total number of habitat types assigned for all 179 parrotfish and surgeonfish species.

Functional classification based on dietary targets and both pre-and post-ingestive processes were determined for each species to more accurately portray the ecological features of these groups and their impact on different coral reef systems. Based on this information, each species was assigned to one of four feeding functional groups: detritivore, herbivore, omnivore, and planktivore [24], [35]. Only four species had no available information on dietary characteristics and represent those in the ‘No Information’ category. Classifications are based on post-ingestive processes, main dietary items, and identification of the main nutrient assimilated by each species. The percentage of feeding modes across all species was calculated by dividing the sum of each classification with the total number of species.

### Coral Reef Loss and Habitat Decline

In light of the susceptibility of parrotfishes and surgeonfishes to habitat degradation, coral reef habitat decline was estimated for each species. The percentage of destroyed and declining coral reef in a species' range was calculated based on estimates of effectively lost reefs and reefs at critical stages in each global geographic node as reported by Wilkinson (2008) [63]. For each species, the percentage of destroyed coral reef and declining coral reef within each species range was calculated using a weighted average based on the amount of coral reef, calculated per species based on WCMC (2010) [94] global coral reef distribution data, within each species' range per geographical node. Estimates of destroyed coral reef in each geographic node are defined as the sum of the percentage of reefs with greater than 90% coral cover loss over at least the past 15 to 20 years that are unlikely to recover [63], [95], while critically declining reef is defined as the percentage of reefs with between 50–90% coral cover loss and is likely to join the total coral loss category within 10 to 20 years [63], [95]. The Global Coral Reef Monitoring Network series [63], [95]–[98] is widely cited for the estimates of global and regional reef status and threats to corals [66], [99]–[102]. However, it is important to note that these estimates only provide regional averages of coral reef destruction and decline, without any quantitative estimate of uncertainty. The averages do not account for variability in the estimates of reef decline and degraded reefs attributed to the range of methods employed in data collection, the scope of reefs covered per region, and the confidence in the methods used to produce the data in coral reef countries and states around the world [63].We recognize the limitations of these estimates, especially as coral reef and fish species are generally not equally distributed across any given geographic region; therefore, coral reef habitat decline can be greater or lower in any particular site across a region.

## Results and Discussion

Of the 179 species assessed, only three qualified for listing under a threatened Category: the Greenback Parrotfish (*Scarus trispinosus)* listed as Endangered; *Bolbometopon muricatum* and Kapingamarangi Surgeonfish (*Acanthurus chronixis*) listed as Vulnerable. Of these three species, two are large-bodied, long-lived and experiencing significant population declines from intense fishing pressure. The third species, *A. chronixis*, has a restricted distribution known only from Kapingamarangi Atoll, Caroline Islands, and is also exploited by subsistence fishers. Two species, Bower's Parrotfish (*Chlorurus bowersi*) and the Yellowtail Parrotfish (*Scarus hypselopterus*), did not fully meet the thresholds and conditions provided in the Criteria for listing under a threatened Category, but were very close to these thresholds and were thus assessed as Near Threatened. A complete list of species, Red List Category and, associated information is provided in Table S1.

The majority of the parrotfishes and surgeonfishes, (86% or 155 species) were listed as Least Concern. These species have broad distributional ranges, high turnover rates, occur in a wide variety of habitats, and are close to the base of the food chain as these species feed on an array of sources that include plant materials, detrital aggregates, bacterial and meiofaunal complexes [24], [25], 32,35,36. These species also maintain high abundances at many sites. Many of the species listed as Least Concern are exploited in artisanal and commercial fisheries in parts of their range and occur in areas where habitat degradation and illegal fishing practices are prevalent. The global population decline over the past three generation lengths for each of these species was, however, below the threshold required (30%) for placement in a threatened Category. Nineteen species (10%) were listed as Data Deficient, seven (37%) of which had specific threats including intense fishing pressure in parts of their ranges. This listing is attributed to the lack of data to adequately quantify the impact of fishing on the species' global populations.

Of special concern is the largest parrotfish in the Atlantic, *Scarus guacamaia*, which was assessed as Data Deficient [103], although was previously listed as Vulnerable (IUCN 1996). This species achieves sizes of over 1 m (TL) and is widely distributed in the western Atlantic: Bermuda, south Florida, and throughout the Caribbean. Records from Brazil are based on a few museum specimens and anecdotal accounts [104]. Although Ferreira *et al.* (2005) [104] suggest that *S. guacamaia* is locally extinct, this species is confirmed as a vagrant along the Brazilian coast. In the Caribbean, this species has experienced significant localized population declines from destruction and loss of its mangrove habitats and historical overfishing [58]. It was historically fished to very low levels throughout its range; however, its populations seem to have stabilized at a small size for the past few generations [105]. Currently *S. guacamaia* appears to occur in high densities only in areas that are protected from fishing [105], a characteristic that is shared by several large-bodied parrotfishes [42] and groupers [106]. The lack of adequate historical population data combined with the rarity of current sightings and subsequent difficulty in coordinating efforts to determine its current population size has resulted in the inability to effectively quantify population declines over time.

However, *S. guacamaia* shares a number of parallel traits with *Bolbometopon muricatum*, the largest parrotfish in the Indo-Pacific. Intrinsic life history characteristics such as large size, natural rarity and shallow foraging areas render both of these species particularly susceptible to overfishing mainly spearfishing. Both species recruit into very shallow water, sheltered reef and mangrove sites that are increasingly impacted by habitat modification and degradation. *Scarus guacamaia* is experiencing >30% decline, destruction of coral reef habitat (which makes up 7% of its range) and is exposed to extensive mangrove deforestation in many parts of its distribution. Unlike *B. muricatum*, which inhabits a wide oceanic basin and could find refuge on isolated oceanic islands in the Indo-Pacific, *S. guacamaia* inhabits a smaller oceanic habitat and may not have access to the types of refuge available for *B. muricatum*. For these reasons, *S. guacamaia* is likely to be conservation dependent, especially as recorded densities are highest only in areas where protection is present. In the absence or cessation of protective measures, *S. guacamaia* may currently qualify for a re-listing of Near Threatened, or possibly one of the threatened categories in the near future (within a period of 5 years).

Also of special concern is *Bolbometopon muricatum*, listed as Vulnerable, which is the largest of all the scarines, reaching 1.4 m (FL). Intrinsic life history characteristics exhibited by *B. muricatum*, such as large body size, nocturnal aggregating behavior in shallow lagoons, reef caves or coral structures, daytime foraging in shallow waters [51], [53], and aggregate spawning [46], make this species particularly vulnerable to overexploitation, especially with the utilization of SCUBA and lamps when spearfishing at nighttime [51]–[54]. This species is iconic on tropical coral reefs of the Indo-Pacific and is one of the most important bioeroder on reefs as well as the largest coral predator [16], [42]. *B. muricatum* is heavily fished in most parts of its wide distributional range and is rare and virtually extinct at some locations (i.e., Guam, Marshall Islands, Fiji, East Africa, Philippines [16], [53], [61]. This species was previously reported to be common to abundant throughout its range, but now appears to be only abundant on isolated oceanic islands, areas where there are no existing fisheries for this species or in areas where stringent conservation policies are in place [42], [53], [107]. A recent study on the demographics of *B. muricatum* indicated that on the Great Barrier Reef, adult and juvenile habitats are spatially separated with juveniles located mainly in inshore areas. The marked spatial separation between recruitment and adult sites on over 5,000 hours of independent observations [42] in one of the world's best marine protected areas suggests that disturbances to juvenile habitats may have major flow-on effects to adult populations even in areas that support high densities of adults. The status of this species appears to be more dependent on maintenance of recruit habitats, as well as protection of schooling and foraging areas [108]. Furthermore, comprehensive surveys of more than a thousand fish census transect surveys in the Philippines demonstrate that this species is almost absent to very rare [61]. There is urgent need therefore, for better assessment of potential recruit habitats, especially in areas within its distribution that are heavily degraded and exploited.

### Regional Assessments

**Figure 1:**
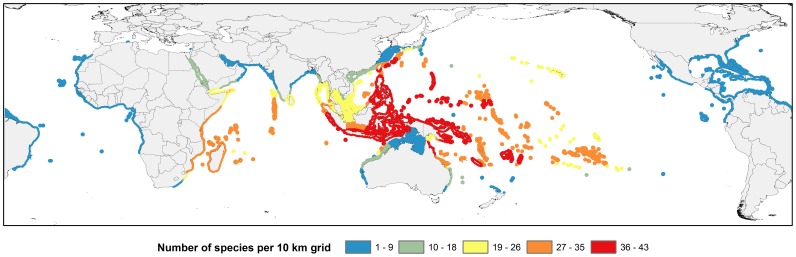
Global species richness patterns. a. Species richness of parrotfishes of the world. b. Species richness of surgeonfishes of the world.

Highest biodiversity for parrotfishes and surgeonfishes is found in the Indo-West Pacific (Figure 1a,b), particularly the Coral Triangle region which encompasses much of Indonesia, Malaysia, the Philippines, Brunei, Timor L'Este, Papua New Guinea, and the Solomon Islands [109]. A total of 105 species of parrotfish and surgeonfish occur in the Exclusive Economic Zones of these six countries, with as many as 76 occurring in the same location. This marine biodiversity hotspot is followed by the eastern and western Indian Ocean and Oceania in terms of absolute number of species.

The Coral Triangle region is well recognized as the center of marine species biodiversity. This pattern of high species concentrations in the region is shared by corals, reef fishes, and invertebrate groups [5], [6], [109]–[111]. The Coral Triangle epicenter of marine biodiversity is also unfortunately known to be highly impacted by a multitude of threats including overfishing, pollution, and rampant habitat modification and loss [61], [109], [112]–[115]. A relatively low proportion (1.7%) of these iconic fish groups is globally listed in threatened Categories. These fishes demonstrate inherent biological characteristics such as broad ranges and commonness that would traditionally be deemed sufficient safeguards against extinction. This is evident in the widely distributed *B. muricatum* [51], [53], listed as Vulnerable, and the common and abundant *Z. flavescens* [76], [78], listed as Least Concern. Nevertheless, these characteristics do not preclude localized population depletions of these species [116].

Parrotfishes and surgeonfishes are important sources of protein in the Coral Triangle region and are commercially targeted with many species prominent in fish markets. Of the 105 species in the Coral Triangle, 18 are commonly targeted commercially in multi-species fisheries, with many more species actually present in catches. Of the commonly targeted species, 13 were at a higher risk of extinction regionally compared to their global assessment (Table 2). For example, Bleeker's Parrotfish (*Chlorurus bleekeri*) is listed as Least Concern globally but is assessed as Near Threatened in the Coral Triangle region. This species is heavily fished throughout the region, with recorded reductions of 50–60% in the Philippines from resource exploitation [59]. In areas where it is exploited, mean size reductions are evident and could lead to a reduction in female reproductive output [117]. In addition to overfishing, *C. bleekeri* also inhabits sheltered coral reef habitats that are under pressure from coastal development, pollution, and climate change [102], [115].

**Table 2:** Regional Red List Assessments of commercially important parrotfishes and surgeonfishes in the Coral Triangle (EN = Endangered, VU = Vulnerable, NT = Near Threatened, LC = Least Concern, DD = Data Deficient).

Family	Genus Species	Global Red List Assessment	Coral Triangle Assessment
Acanthuridae	*Acanthurus lineatus*	LC	NT
Acanthuridae	*Naso lituratus*	LC	NT
Acanthuridae	*Naso lopezi*	LC	DD
Acanthuridae	*Naso mcdadei*	LC	DD
Acanthuridae	*Naso unicornis*	LC	NT
Acanthuridae	*Paracanthurus hepatus*	LC	DD
Scarinae	*Bolbometopon muricatum*	VU	EN
Scarinae	*Chlorurus bleekeri*	LC	NT
Scarinae	*Chlorurus bowersi*	NT	NT
Scarinae	*Chlorurus japanensis*	LC	NT
Scarinae	*Scarus dimidiatus*	LC	NT
Scarinae	*Scarus flavipectoralis*	LC	NT
Scarinae	*Scarus forsteni*	LC	NT
Scarinae	*Scarus ghobban*	LC	NT
Scarinae	*Scarus hypselopterus*	NT	NT
Scarinae	*Scarus niger*	LC	NT
Scarinae	*Scarus quoyi*	LC	NT
Scarinae	*Scarus rivulatus*	LC	NT

Additional regional assessments are needed to better understand the conservation status of these species, thereby bringing to the forefront significant threats to the local populations that would not otherwise be captured at the global scale. A comprehensive regional assessment for all known species of marine bony and cartilaginous shorefishes, corals, mangroves, and seagrasses was conducted for the Tropical Eastern Pacific (TEP) [82]. For the five parrotfishes and surgeonfishes that are endemic to the TEP, regional TEP Red List assessments are the same as global assessments. However, regional assessments were not conducted for the remaining six species that occur in the TEP but have distributions that span much of the Indo-Pacific. Priority areas for additional regional assessments include the Caribbean, Oceania and the Indian Ocean, where fishing is thought to be heavily impacting a number of parrotfishes and surgeonfishes.

### Habitat Status and Decline of Coral Reefs

**Figure 2:**
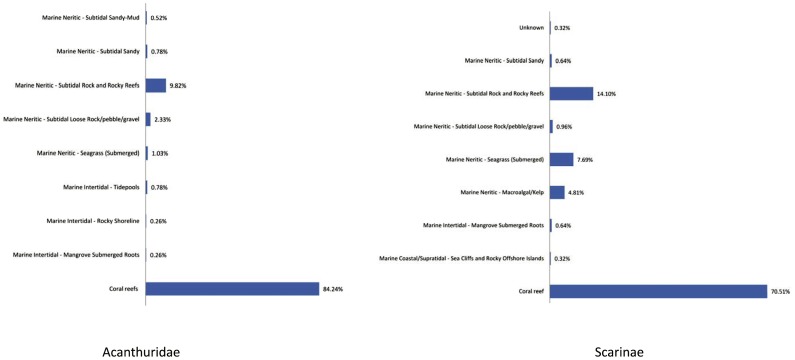
Percentage of parrotfishes and surgeonfishes in each habitat type.

The adverse effects of coral loss and habitat degradation (including declines in species abundance and diversity, reduced physiological condition, decreased settlement, change in community structure, etc) [64], [65], [118]–[120] on species dependent upon coral reefs for food and habitat have been well documented [26], [64], [65], [67], [73], [99], [102], [121]–[123]. The majority of parrotfishes and surgeonfishes (140 species or 78%) inhabit coral reefs, but many can also be found in a variety of other habitats (Figure 2). Less than half of all parrotfishes and surgeonfishes (78 species or 44%) exclusively inhabit coral reef habitats, and the majority of these (72 species) are listed as Least Concern, indicating that information on habitat loss alone was not enough to definitively estimate significant species population decline at a global level. However, of the species that are exclusive to coral reefs, it is important to note that 64 species (82%) are experiencing greater than 30% coral reef area loss and decline in reef habitat quality (Table S2). Coral reef area loss and decline was estimated to be present within almost all the 179 species' ranges, however, there is variation in the reliance of different species on coral reefs based on species' habitat preferences (i.e., those species that spend the majority of their life stages on coral reef habitat vs. others that primarily utilize seagrass beds, mangroves, algal beds, and rocky reefs). For example, there is an estimated 85% coral reef area loss and degradation within the range of *Scarus persicus*, but as a mixed habitat species known to inhabit rocky reefs and coral patches, it is not know to what extent it may actually be affected by coral reef loss (Table S2). By contrast, the exclusively coral reef dependent Spot-fin Parrotfish (*Scarus maculipinna*), known only from reefs off of Thailand and Indonesia, may be significantly impacted by an estimated 60% coral reef habitat loss and decline within its range. However, this species was assessed as Data Deficient, as it was fairly recently described in 2007, and there is very limited information available on population status, abundance, or life history characteristics.

**Figure 3:**
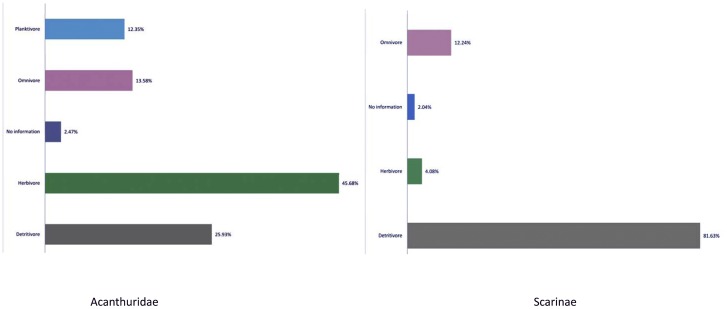
Percentage of destroyed and declining reef in each species' range vs Red List Category. Center line = median value, box boundaries = 25^th^ and 75^th^ percentiles, whiskers = 10^th^ and 90^th^ percentiles, black dots = outliers.

The three threatened and Near Threatened species that are exclusive to coral reefs have a lower average proportion of destroyed and declining reef area within their ranges compared to those listed as Least Concern or Data Deficient (Figure 3). This low proportion may be attributed to the limited range distribution of one species (*A. chronixis*). However, *Chlorurus bowersi*, listed as Near Threatened, has a relatively restricted range in the Indo-Pacific and is subject to intense fishing pressure in over 90% of its distribution. Fishing coupled with rampant habitat loss may prove detrimental to this species in the near future and warrant listing in a higher threat category. Interestingly, some Data Deficient and Least Concern species have as much as 60% coral reef decline and area loss within their range. These species currently listed in low extinction risk categories may necessitate listing at a higher level of threat as habitat loss and degradation persist within the species' range. Models of extrinsic threat factors may be used to predict probability of extinction in the future. Indeed, Davidson et al. (2012) [124] show that of the 40% (46 species) of marine mammals currently listed in Data Deficient category, 28% (13 species) were identified to be at a higher risk of extinction based on intrinsic and extrinsic predictor variables of threat.

Many parrotfishes and surgeonfishes exhibit strong associations with coral reefs, especially as live coral provide suitable habitat for juveniles of both groups, and several sub-adult and adult species of surgeonfishes are primarily associated with coral [125], [126]. For example, juveniles of *Z. flavescens* recruit into live branching corals and areas of high coral cover [78]. A recent study of a coral-dominated reef in the Indo-Pacific highlighted the importance of protecting juvenile habitats such as mangroves, seagrasses, and coral reefs. This study indicated that maintaining pristine primary habitats would be beneficial to the population status of fishes that demonstrate ontogenetic shifts in habitat preference [127]. Furthermore, the combined effects of over-exploitation and habitat degradation have been identified as significant agents of population declines and species extinctions [61], [69], [75], [128], [129], with the ability to alter the structure of coral reef fish communities [65], [69].

Widespread coral reef loss and declining habitat conditions are particularly worrying for some corallivorous excavating parrotfishes, particularly *B. muricatum*, the Bicolor Parrotfish (*Cetoscarus bicolor*), and other species of the genus *Chlorurus* [16], [42], [130]. These large excavating parrotfishes play major roles in ecosystem processes such as bioerosion and coral predation [42]. On pristine reef systems, erosion by physical and/or biological agents is primarily attributed to the feeding activity of parrotfishes [16], [41], [131]. Bioerosion by parrotfishes is a major process in Indo-Pacific coral reef ecosystems, with erosion rates often matching maximum estimated calcification rates [16]. The absence of these species of considerable ecological importance will have major impacts on the overall health of coral reefs [16], [42], [132].

### Feeding Guilds and Functional Ecology

Studies on the trophic ecology of herbivorous fishes have focused on the ecological impacts of these fishes on reefs, in particular, the contributions of herbivores in the prevention of macro-algal phase shifts in coral reef systems [14], [24], [28], [29], [122], [132], [133]. Other studies have shown that the loss of large bioeroding parrotfishes may include major shifts in ecosystem dynamics, from steady-state calcification to carbonate accumulation [16], changes in species composition of coral reefs to favor faster growing coral species [42], and structural instability of coral reef systems attributed to storms and echinoid invasions [16]. However, in general, the effects of the loss of parrotfishes and surgeonfishes to coral reef ecosystems, at the site or global scale, are complex and poorly understood. This study takes a different approach in assessing the ecological contributions of these nominally recognized herbivores through examination of the underlying factors that are attributed to the iconic roles the parrotfishes and surgeonfishes perform on coral reefs. Herbivory is defined by post-ingestive processes through examination of alimentary tract contents, algal components degraded along the gut, and measurement of short-chain fatty acid (SCFA) production in the hindgut [24], [25], [32]–[36]. This type of species-specific information on nutritional ecology is essential in building a better understanding of how specific roles in these groups will affect coral reef systems. This is especially important in the face of rapid and recurring climate change events [28], [36], and will be vital for concentrating conservation efforts globally.

**Figure 4:**
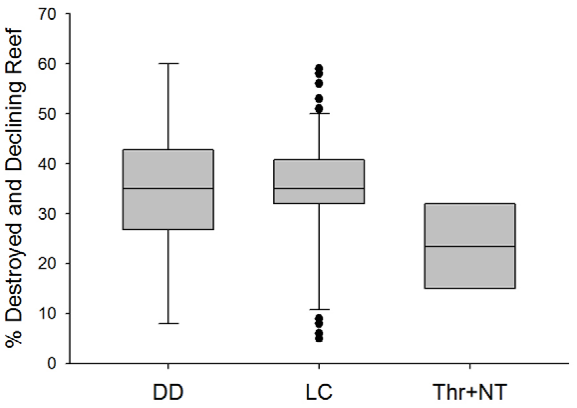
Feeding classification of parrotfishes and surgeonfishes based on dietary targets and post-digestive processes.

There is great diversity in feeding patterns exhibited by parrotfishes and surgeonfishes [13]. Examination of functional classification based on dietary targets and both pre- and post-ingestive processes for all species reveal four functional groups: detritivore, herbivore, omnivore, and planktivore (Figure 4). The majority of species in both groups (58%) are detritivores, ingesting detritus/bacterial complexes, meiofauna, coral tissue, and sessile invertebrates. The detritivores are represented by grazing surgeonfishes and the two groups of parrotfishes: excavators and scrapers [130]. Despite the assumed notion of herbivory by the parrotfishes and surgeonfishes, only 21% of the species are strictly herbivores, with living plant material, primarily algae, as the dominant dietary source and carbohydrates as the main assimilated macronutrient [25]. The remaining species were classified as omnivores (13%) that ingest macroscopic algae, detrital material, and benthic invertebrates. Planktivores (6%) include members that are obligate feeders on plankton, such as *Acanthurus thompsoni* and *A. mata*. Compared to herbivores and detritivores, members of the last two categories have limited impact on the benthic biota of the reef. Four of the five species listed in threatened or Near Threatened Categories are classified as detritivores.

### Major Threats and Fisheries

**Figure 5:**
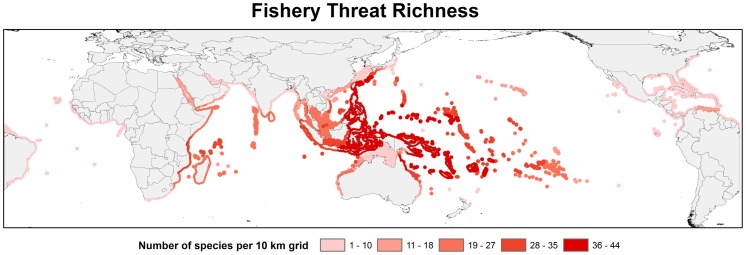
Number of species threatened by fisheries.

Of all the threats identified for parrotfishes and surgeonfishes, none compete with the pervasive and deleterious effects of fishing. At least 40% (73 species) were determined to be impacted by either small-scale or large-scale fisheries. Other threats affecting parrotfishes and surgeonfishes include habitat modification and degradation (6%), pollution (3%), or by-catch (1%) (Table S3). The Coral Triangle region has the highest number of species impacted by fisheries (Figure 5), followed by Oceania and the Western Indian Ocean. Regional assessments are needed for these species in both Oceania and the Western Indian Ocean to better understand the impacts of fishing on regional populations.

The effects of overfishing on the loss of large consumer species in marine ecosystems have been well documented [60], [61], [121], [128], [134]–[139]. Fishing and over-exploitation has led to localized declines of several of the large species of parrotfish including *S. guacamaia* [58], [104], *B. muricatum* [16], [42], [46], [53], [107], *C. bowersi* [59], and *S. trispinosus* [140]. In coral reef systems, larger-bodied food fish in general (e.g. groupers, snappers, and wrasses) are becoming increasingly rare [23], [56], [106], [141], [142], with high exploitation rates partly attributed to the lucrative benefits of the live reef fish trade [106], [143], [144] and to the relative ease of capture of these reef fishes [51], [52]. The removal of the top predators (e.g., sharks, groupers, snappers, jacks, and wrasses) has led to a shift in target preference to herbivorous fishes and planktivores [26], [50], [53], [136], [145] and increased abundance in catch of parrotfishes [146]–[148].

Changes in size structures are key indicators of the effects of human disturbance [137], [142], [149], [150], and can be used as a predictor of the vulnerability of coral reef fishes to overfishing [137], [151]. Indeed, in a parrotfish artisanal fishery in Western Solomon Islands, the decreasing trend in Catch Per Unit Effort (CPUE) suggests that once the larger species have declined, e.g. Ember Parrotfish (*Scarus rubroviolaceus*), Steephead Parrotfish, *Chlorurus microrhinos*), Blue-barred Parrotfish (*Scarus ghobban*), and *B. muricatum*, the target is shifted to the smaller scraping species, e.g. Yellowbarred Parrotfish (*Scarus dimidiatus*), Globehead Parrotfish (*Scarus globiceps*), and Yellowband Parrotfish (*Scarus schlegeli*) [56].

### Conservation Actions in Place

**Figure 6:**
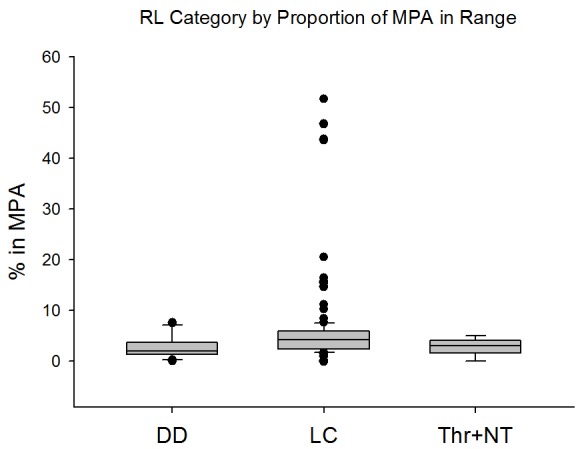
Red List Category by Proportion of MPA in each species' range. Center line = median value, box boundaries = 25^th^ and 75^th^ percentiles, whiskers = 10^th^ and 90^th^ percentiles, black dots = outliers.

The principal conservation actions in place for parrotfishes and surgeonfishes are presence in marine reserves, and fisheries restrictions and limits. Marine reserves have been shown to facilitate recovery of populations of these key functional groups [27], [45], [59], [152]–[155]. Indeed, marine reserves have been successful in protecting some species from further population declines (*Z. flavescens* in Hawaii [155], *Acanthurus spp.* and *Naso spp.* in the Philippines [152], and *B. muricatum* in Australia [16]). However, the vast majority of parrotfishes and surgeonfishes have less than 5% of their range within a marine protected area (Figure 6), and most often the actual level of protection within the marine protected area is unknown. There are no differences among species in terms of Red List Category and average proportion of range within a marine protected area. However, several relatively small-ranging species such as *Calotomus zonarchus* from the Hawaiian Islands and *Prionurus microlepidotus* from eastern Australia, both listed as Least Concern, currently have a large proportion (>45%) of their range within a protected area. By contrast, *Acanthuris chronixis*, listed as Vulnerable, is not protected within any marine protected area within its known range in the Caroline Islands.

Because of the long recognized ecological services provided by the parrotfishes and surgeonfishes, species-specific management measures are in place in Bermuda, wherein all species of parrotfishes are protected under the Fisheries (Protected Species) Order 1978 [156]. In addition, there have been recent protective measures in place for the parrotfishes in Belize, wherein the fishing of grazers, defined as any scarinae species and Acanthuridae species [157], is prohibited. In the Turks and Caicos, the fishing and selling of any species of parrotfish is prohibited [158], and the Caribbean Management Council, which comprises the Commonwealth of Puerto Rico and United States Virgin Islands, has prohibited the harvest and possession of Midnight Parrotfish (*Scarus coelestinus*), Blue Parrotfish (*Scarus coeruleus*), and *S. guacamaia* as well as reduced parrotfish harvest in St. Croix [159].

### Conclusions and Recommendations

Parrotfishes and surgeonfishes are critical components of coral reefs, a habitat that is experiencing extensive global decline [63], [102], [115], and both families play vital ecological roles in coral reef trophodynamics. Although only three of the 179 species are currently considered to be at elevated risk of extinction, significant threats including fishing and habitat loss are contributing to localized population declines, particularly in the Coral Triangle. Additional comprehensive regional assessments are needed in areas of high fishing pressure, such as Oceania and the Western Indian Ocean. With better habitat, fisheries, and population information, many species currently listed as Data Deficient may indeed qualify for a threatened category in the near future. In particular, any large-bodied fish with little formalized protection (such as *Bolbometopon muricatum*, *Scarus guacamaia*, etc.) that are present in areas of high fishing pressure and or high coral reef habitat loss, should be a priority for further research and monitoring, regardless of their IUCN Red List Category.

It is important to note that despite the prevalence of marine reserves, the effectiveness of these conservation efforts, is rarely measured, and enforcement is often weak or absent in many parts of the world. Urgent protection and effective protective legislation is needed as well as continued monitoring of harvest levels and population status, especially for those species already at increased risk of extinction. In addition to scaling up of species-specific conservation actions, existing conservation practices need to take into account the reproductive strategies of these species that render them vulnerable to extinction. Parrotfishes and surgeonfishes form spawning aggregations with at least 14 and nine confirmed aggregations for the Acanthuridae and scarinae respectively [160]. There are many more species of surgeonfishes that are known to aggregate where they occur. In addition, aggregation (feeding and spawning) is a widely recognized attribute of this family [2], [161]. In view of the economic and ecological services that these species provide, protection of aggregation sites for these species is imperative. Protection of aggregation sites is particularly important for *B. muricatum*, as the schooling behavior of this species in shallow waters makes it highly vulnerable to exploitation [108].

In order to afford adequate conservation of these species, critical knowledge gaps also need to be addressed. Although a number of demographic, trophodynamic, distribution, and habitat association studies have been conducted for both groups, there is a general lack of information, on life history, dietary requirements, fisheries information and trade data for most of the commercially targeted coral reef fishes [106]. Specifically, the effects of overexploitation on the large-bodied parrotfishes and surgeonfishes and the inter-specific relationships of the loss of the larger species that provide crucial ecological benefits to coral reefs need further study. The effects of widespread and prevalent coral reef loss and habitat degradation also need to be further understood, especially for the species that exclusively depend on this habitat. In addition, the factors that underpin the iconic ecological roles that these species provide, such as their varied feeding strategies, need to be better understood, and their specific impacts to coral reef systems, further studied. Finally, it is important to focus conservation and management efforts on ecologically and economically important species, and to persist on building a strong knowledge base to counter the effects of biodiversity loss in our rapidly changing world.

## Supporting Information

Table S1 **Complete list of species, Red List Category and associated information on life history, dietary classification and presence in marine reserves.**
(PDF)

Table S2 **Percentage of coral reef loss and declining reef area for all species of parrotfishes and surgeonfishes (1 = exclusively coral reef dependent, 2 = primarily found in coral reefs, 3 = mixed habitat).**
(PDF)

Table S3 **Major threats identified for each species of parrotfish and surgeonfish.**
(PDF)
