# Supplementary material for: The Likelihood of Extinction of Iconic and Dominant Herbivores and Detritivores of Coral Reefs: The Parrotfishes and Surgeonfishes
Source: PLoS One. 2012 Jul 11;7(7):e39825. doi: 10.1371/journal.pone.0039825 (PMC3394754; doi:10.1371/journal.pone.0039825)
Supplement: Table S1 — Complete list of species, Red List Category and associated information on life history, dietary classification and presence in marine reserves. (PDF) [file pone.0039825.s001.pdf]

Family	Scientific name	Red List Category	Maximum size	Longevity (years)	Dietary classification	% of species range in MPA
Acanthuridae	*Acanthurus blochii*	LC	45 (SL)	35	Detritivore	6.18
Acanthuridae	*Acanthurus dussumieri*	LC	54 (TL)	28	Detritivore	5.13
Acanthuridae	*Acanthurus fowleri*	LC	45 (TL)		Detritivore	1.96
Acanthuridae	*Acanthurus grammoptilus*	LC	35 (TL)		Detritivore	11.22
Acanthuridae	*Acanthurus leucocheilus*	LC	48 (TL)		Detritivore	2.07
Acanthuridae	*Acanthurus maculiceps*	LC	40 (TL)		Detritivore	3.61
Acanthuridae	*Acanthurus nigricauda*	LC	40 (TL)		Detritivore	4.17
Acanthuridae	*Acanthurus olivaceus*	LC	35 (TL)	33	Detritivore	6.02

Acanthuridae	*Acanthurus pyroferus*	LC	29 (TL)	28	Detritivore	4.73
Acanthuridae	*Acanthurus reversus*	LC	34 (SL)		Detritivore	0.00
Acanthuridae	*Acanthurus tennentii*	LC	31 (TL)		Detritivore	1.73
Acanthuridae	*Acanthurus tristis*	LC	25 (TL)		Detritivore	2.50
Acanthuridae	*Ctenochaetus binotatus*	LC	22 (TL)	25	Detritivore	4.32
Acanthuridae	*Ctenochaetus cyanocheilus*	LC	17.7 (TL)		Detritivore	5.36
Acanthuridae	*Ctenochaetus flavicauda*	LC	11.8 (SL)		Detritivore	5.16
Acanthuridae	*Ctenochaetus hawaiiensis*	LC	28.1 (TL)		Detritivore	4.43
Acanthuridae	*Ctenochaetus marginatus*	LC	27 (TL)	15	Detritivore	15.74

Acanthuridae	*Ctenochaetus striatus*	LC	26 (TL)	36	Detritivore	3.81
Acanthuridae	*Ctenochaetus strigosus*	LC	19 (TL)		Detritivore	43.81
Acanthuridae	*Ctenochaetus tominiensis*	LC	17.5 (TL)	20	Detritivore	2.90
Acanthuridae	*Ctenochaetus truncatus*	LC	18.5 (TL)		Detritivore	1.71
Labridae	*Bolbometopon muricatum*	VU	140 (FL)	40	Detritivore	5.09
Labridae	*Cetoscarus bicolor*	LC	50 (TL)	25	Detritivore	2.79
Labridae	*Cetoscarus ocellatus*	LC	>70 (TL)	25	Detritivore	4.99
Labridae	*Chlorurus atrilunula*	LC	36 (TL)		Detritivore	1.51
Labridae	*Chlorurus bleekeri*	LC	49 (TL)	14	Detritivore	5.33

Labridae	*Chlorurus bowersi*	NT	35 (TL)	14	Detritivore	3.07
Labridae	*Chlorurus capistratoides*	LC	40 (TL)		Detritivore	2.75
Labridae	*Chlorurus cyanescens*	LC	50 (TL)		Detritivore	1.83
Labridae	*Chlorurus enneacanthus*	LC	60 (TL)		Detritivore	3.07
Labridae	*Chlorurus frontalis*	LC	50 (TL)		Detritivore	6.76
Labridae	*Chlorurus genazonatus*	LC	26 (TL)		Detritivore	2.38
Labridae	*Chlorurus gibbus*	LC	70 (TL)		Detritivore	2.75
Labridae	*Chlorurus japanensis*	LC	24 (TL)		Detritivore	5.21
Labridae	*Chlorurus microrhinos*	LC	80 (TL)	17	Detritivore	5.12

Labridae	*Chlorurus oedema*	LC	45 (TL)		Detritivore	2.03
Labridae	*Chlorurus perspicillatus*	LC	62 (TL)		Detritivore	43.64
Labridae	*Chlorurus rhakoura*	LC	50 (TL)		Detritivore	2.09
Labridae	*Chlorurus sordidus*	LC	39 (TL)	9	Detritivore	1.90
Labridae	*Chlorurus spilurus*	LC	25-37 (TL)	15	Detritivore	5.78
Labridae	*Chlorurus strongylocephalus*	LC	45-70 (TL)	9 (males); 8 (females)	Detritivore	1.67
Labridae	*Chlorurus troschelii*	LC	40 (TL)		Detritivore	2.47
Labridae	*Hipposcarus harid*	LC	75 (TL)		Detritivore	1.94
Labridae	*Hipposcarus longiceps*	LC	60 (TL)	12	Detritivore	5.04

Labridae	*Scarus altipinnis*	LC	60 (TL)	13	Detritivore	7.09
Labridae	*Scarus arabicus*	LC	>50 (TL)	25	Detritivore	0.15
Labridae	*Scarus caudofasciatus*	LC	50 (TL)		Detritivore	2.41
Labridae	*Scarus chameleon*	LC	25 (TL)	6	Detritivore	6.29
Labridae	*Scarus chinensis*	DD			Detritivore	1.38
Labridae	*Scarus coelestinus*	DD	77 (TL)		Detritivore	7.57
Labridae	*Scarus coeruleus*	LC	90 (TL)	16	Detritivore	7.37
Labridae	*Scarus collana*	LC	33 (TL)		Detritivore	2.79
Labridae	*Scarus compressus*	LC	68 (TL)		Detritivore	6.57

Labridae	*Scarus dimidiatus*	LC	40 (TL)		Detritivore	4.66
Labridae	*Scarus dubius*	LC			Detritivore	43.64
Labridae	*Scarus falcipinnis*	LC	50 (TL)	10	Detritivore	1.36
Labridae	*Scarus ferrugineus*	LC	50 (TL)	15	Detritivore	1.57
Labridae	*Scarus festivus*	LC	45 (TL)		Detritivore	1.85
Labridae	*Scarus flavipectoralis*	LC	30 (TL)	5	Detritivore	5.40
Labridae	*Scarus forsteni*	LC	55 (TL)		Detritivore	4.07
Labridae	*Scarus frenatus*	LC	47 (TL)	8 (Indian Ocean) and 20 (Pacific Ocean)	Detritivore	4.16
Labridae	*Scarus fuscocaudalis*	LC	25 (TL)		Detritivore	2.20

Labridae	*Scarus fuscopurpureus*	LC	38 (TL)		Detritivore	1.76
Labridae	*Scarus ghobban*	LC	75 (TL)	13	Detritivore	1.95
Labridae	*Scarus globiceps*	LC	27 (TL)		Detritivore	4.80
Labridae	*Scarus guacamaia*	DD	120 (TL)	16	Detritivore	7.67
Labridae	*Scarus hoefleri*	LC	60 (TL)		Detritivore	1.59
Labridae	*Scarus hypselopterus*	NT	31 (TL)	10	Detritivore	2.23
Labridae	*Scarus iseri*	LC	35 (TL)	8	Detritivore	7.57
Labridae	*Scarus koputea*	LC	31 (TL)		Detritivore	0.00
Labridae	*Scarus longipinnis*	LC	30 (TL)	5-7	Detritivore	16.47

Labridae	*Scarus niger*	LC	32-40 (TL)	23	Detritivore	3.40
Labridae	*Scarus obishime*	DD	70 (SL)		Detritivore	0.34
Labridae	*Scarus oviceps*	LC	30 (TL)		Detritivore	5.50
Labridae	*Scarus ovifrons*	DD	78 (TL)		Detritivore	2.25
Labridae	*Scarus perrico*	LC	80 (TL)		Detritivore	6.55
Labridae	*Scarus persicus*	LC	56 (TL)	22	Detritivore	2.12
Labridae	*Scarus prasiognathos*	LC	70 (TL)		Detritivore	2.20
Labridae	*Scarus psittacus*	LC	30 (TL)	5	Detritivore	5.12
Labridae	*Scarus quoyi*	LC	40 (TL)		Detritivore	2.26

Labridae	*Scarus rivulatus*	LC	40 (SL)	11-12	Detritivore	4.74
Labridae	*Scarus rubroviolaceus*	LC	70 (TL)	15	Detritivore	5.30
Labridae	*Scarus russelii*	LC	51 (TL)		Detritivore	2.05
Labridae	*Scarus scaber*	LC	36 (TL)		Detritivore	1.94
Labridae	*Scarus schlegeli*	LC	31	8	Detritivore	4.02
Labridae	*Scarus spinus*	LC	30 (TL)		Detritivore	4.82
Labridae	*Scarus taeniopterus*	LC	35 (TL)	9	Detritivore	7.55
Labridae	*Scarus tricolor*	LC	26.6 (SL)		Detritivore	3.71
Labridae	*Scarus trispinosus*	EN	70 (TL)		Detritivore	3.85

Labridae	*Scarus vetula*	LC	50 (FL)	20	Detritivore	7.17
Labridae	*Scarus viridifucatus*	LC	26 (TL)		Detritivore	1.85
Labridae	*Scarus xanthopleura*	LC	52 (TL)		Detritivore	1.88
Labridae	*Scarus zelindae*	DD	33.2 (SL)	12	Detritivore	3.85
Labridae	*Scarus zufar*	DD	52 (TL)	9	Detritivore	0.14
Labridae	*Sparisoma amplum*	LC	39 (SL)	9-14	Detritivore	3.28
Labridae	*Sparisoma atomarium*	LC	25 (TL)	3	Detritivore	6.65
Labridae	*Sparisoma frondosum*	DD	34.5 (SL)	12	Detritivore	3.62
Labridae	*Sparisoma griseorubrum*	DD			Detritivore	0.25

Labridae	*Sparisoma rubripinne*	LC	47.8 (TL)	7	Detritivore	6.08
Labridae	*Sparisoma tuiupiranga*	LC	15.4 (SL)		Detritivore	3.47
Labridae	*Sparisoma viride*	LC	50 (TL)	14	Detritivore	7.17
Labridae	*Cryptotomus roseus*	LC	13 (TL)		Herbivore	6.50
Acanthuridae	*Acanthurus achilles*	LC	25 (TL)		Herbivore	6.78
Acanthuridae	*Acanthurus auranticavus*	LC	30 (TL)	30	Herbivore	4.43
Acanthuridae	*Acanthurus bahianus*	LC	36 (TL)	32	Herbivore	3.04
Acanthuridae	*Acanthurus bariene*	LC	42 (TL)		Herbivore	3.15
Acanthuridae	*Acanthurus chirurgus*	LC	34.5 (TL)	30	Herbivore	5.68

Acanthuridae	*Acanthurus chronixis*	VU	21.1 (SL)		Herbivore	0.00
Acanthuridae	*Acanthurus coeruleus*	LC	39 (TL)	43	Herbivore	6.29
Acanthuridae	*Acanthurus guttatus*	LC	26 (TL)		Herbivore	6.27
Acanthuridae	*Acanthurus japonicus*	LC	21 (TL)		Herbivore	3.20
Acanthuridae	*Acanthurus leucopareius*	LC	24 (TL)		Herbivore	6.56
Acanthuridae	*Acanthurus leucosternon*	LC	23 (TL)		Herbivore	1.83
Acanthuridae	*Acanthurus lineatus*	LC	38 (TL)	43	Herbivore	4.11
Acanthuridae	*Acanthurus nigricans*	LC	21.3 (TL)	34	Herbivore	6.39
Acanthuridae	*Acanthurus nigrofuscus*	LC	21 (TL)	16	Herbivore	4.75

Acanthuridae	*Acanthurus nigroris*	LC	25 (TL)		Herbivore	43.81
Acanthuridae	*Acanthurus nigros*	LC	22 (TL)		Herbivore	7.00
Acanthuridae	*Acanthurus polyzona*	DD	20 (TL)		Herbivore	1.56
Acanthuridae	*Acanthurus sohal*	LC	40 (TL)		Herbivore	1.69
Acanthuridae	*Acanthurus triostegus*	LC	27 (TL)		Herbivore	5.42
Acanthuridae	*Naso brachycentron*	LC	90 (TL)	31	Herbivore	4.14
Acanthuridae	*Naso elegans*	LC	36 (TL)	17	Herbivore	1.91
Acanthuridae	*Naso lituratus*	LC	37 (FL)	39	Herbivore	5.38
Acanthuridae	*Naso tonganus*	LC	60 (TL)		Herbivore	5.22

Acanthuridae	*Naso tuberosus*	DD	60 (SL)	25	Herbivore	1.29
Acanthuridae	*Naso unicornis*	LC	70 (FL)	58	Herbivore	4.78
Acanthuridae	*Prionurus chrysurus*	DD	30 (SL)		Herbivore	3.06
Acanthuridae	*Prionurus laticlavius*	LC	60 (TL)		Herbivore	20.56
Acanthuridae	*Prionurus maculatus*	LC	45 (TL)		Herbivore	10.28
Acanthuridae	*Prionurus punctatus*	LC	60 (TL)		Herbivore	2.31
Acanthuridae	*Prionurus scalprum*	DD	50 (TL)		Herbivore	2.06
Acanthuridae	*Zebrasoma desjardinii*	LC	40 (TL)		Herbivore	1.85
Acanthuridae	*Zebrasoma flavescens*	LC	20 (TL)	41	Herbivore	8.48

Acanthuridae	*Zebrasoma gemmatum*	DD	23 (TL)		Herbivore	1.53
Acanthuridae	*Zebrasoma rostratum*	DD	26.5 (TL)		Herbivore	6.52
Acanthuridae	*Zebrasoma scopas*	LC	21.5 (TL)	33	Herbivore	3.92
Acanthuridae	*Zebrasoma veliferum*	LC	40 (TL)	30	Herbivore	5.52
Acanthuridae	*Zebrasoma xanthurum*	LC	25.1 (TL)	11	Herbivore	2.49
Labridae	*Leptoscarus vaigiensis*	LC	35 (TL)		Herbivore	4.87
Labridae	*Sparisoma chrysopterum*	LC	46 (TL)	5	Herbivore	7.68
Labridae	*Sparisoma radians*	LC	20 (TL)		Herbivore	6.00
Acanthuridae	*Acanthurus gahhm*	DD	40 (TL)		No info	1.68

Acanthuridae	*Prionurus biafraensis*	LC	24 (TL)		No info	0.12
Labridae	*Scarus maculipinna*	DD	25 (TL)		No info	1.93
Labridae	*Scarus pyrrostethus*	LC	70-90 (TL)	14	No info	4.90
Acanthuridae	*Acanthurus mata*	LC	50 (TL)	23	Omnivore	3.98
Acanthuridae	*Acanthurus monroviae*	LC	45 (TL)		Omnivore	1.72
Acanthuridae	*Acanthurus xanthopterus*	LC	70 (TL)	34	Omnivore	5.18
Acanthuridae	*Naso annulatus*	LC	100 (TL)	23	Omnivore	4.29
Acanthuridae	*Naso brevirostris*	LC	60 (FL)	25	Omnivore	4.89
Acanthuridae	*Naso fageni*	LC	80 (TL)		Omnivore	2.51

Acanthuridae	*Naso maculatus*	LC	60 (TL)	Omnivore	14.68
Acanthuridae	*Naso mcdadei*	LC	75 (SL)	Omnivore	7.49
Acanthuridae	*Naso reticulatus*	DD	57 (TL)	Omnivore	6.24
Acanthuridae	*Naso vlamingii*	LC	60 (TL)	Omnivore	4.27
Acanthuridae	*Prionurus microlepidotus*	LC	70 (TL)	Omnivore	51.72
Labridae	*Calotomus carolinus*	LC	50 (TL)	Omnivore	5.56
Labridae	*Calotomus japonicus*	LC	39 (TL)	Omnivore	3.50
Labridae	*Calotomus spinidens*	LC	30 (TL)	Omnivore	2.96
Labridae	*Calotomus viridescens*	LC	21 (SL)	Omnivore	3.96

Labridae	*Calotomus zonarchus*	LC	33 (SL)		Omnivore	46.78
Labridae	*Nicholsina collettei*	LC	24 (SL)		Omnivore	1.08
Labridae	*Nicholsina denticulata*	LC	35 (TL)		Omnivore	5.00
Labridae	*Nicholsina usta*	LC	30 (TL)		Omnivore	5.53
Labridae	*Sparisoma aurofrenatum*	LC	28 (TL)	7	Omnivore	7.13
Labridae	*Sparisoma axillare*	DD	37 (SL)		Omnivore	3.28
Labridae	*Sparisoma cretense*	LC	50 (TL)		Omnivore	3.39
Labridae	*Sparisoma strigatum*	LC	45 (SL)	10	Omnivore	2.30
Acanthuridae	*Acanthurus albipectoralis*	LC	33 (TL)		Planktivore	15.45

Acanthuridae	*Acanthurus nubilus*	LC	26 (TL)		Planktivore	3.75
Acanthuridae	*Acanthurus thompsoni*	LC	27 (TL)		Planktivore	4.94
Acanthuridae	*Naso caeruleacauda*	LC	30 (TL)		Planktivore	3.27
Acanthuridae	*Naso caesius*	LC	45.6 (SL)		Planktivore	6.95
Acanthuridae	*Naso hexacanthus*	LC	75 (FL)	44	Planktivore	4.88
Acanthuridae	*Naso lopezi*	LC	60 (TL)		Planktivore	2.78
Acanthuridae	*Naso minor*	LC	22.5 (TL)	5	Planktivore	3.93
Acanthuridae	*Naso thynnoides*	LC	35 (TL)	4-5	Planktivore	2.74
Acanthuridae	*Paracanthurus hepatus*	LC	26 (TL)		Planktivore	4.74
