# Supplementary material for: The Likelihood of Extinction of Iconic and Dominant Herbivores and Detritivores of Coral Reefs: The Parrotfishes and Surgeonfishes
Source: PLoS One. 2012 Jul 11;7(7):e39825. doi: 10.1371/journal.pone.0039825 (PMC3394754; doi:10.1371/journal.pone.0039825)
Supplement: Table S2 — Percentage of coral reef loss and declining reef area for all species of parrotfishes and surgeonfishes (1 = exclusively coral reef dependent, 2 = primarily found in coral reefs, 3 = mixed habitat). (PDF) [file pone.0039825.s002.pdf]

Family	Scientific name	Global Red Listing	TOTAL % Destroyed Reef in Range	TOTAL % Destroyed and Declining Reef in Range	Habitat Categories
Acanthuridae	*Acanthurus achilles*	LC	6%	16%	2
Acanthuridae	*Acanthurus albipectoralis*	LC	3%	12%	1
Acanthuridae	*Acanthurus auranticavus*	LC	25%	40%	1
Acanthuridae	*Acanthurus bahianus*	LC	no data	no data	3
Acanthuridae	*Acanthurus bariene*	LC	25%	41%	1
Acanthuridae	*Acanthurus blochii*	LC	19%	33%	2
Acanthuridae	*Acanthurus chirurgus*	LC	14%	37%	2
Acanthuridae	*Acanthurus chronixis*	VU	8%	15%	1
Acanthuridae	*Acanthurus coeruleus*	LC	14%	37%	3
Acanthuridae	*Acanthurus dussumieri*	LC	21%	36%	1
Acanthuridae	*Acanthurus fowleri*	LC	32%	50%	1
Acanthuridae	*Acanthurus gahhm*	DD	4%	8%	1
Acanthuridae	*Acanthurus grammoptilus*	LC	3%	11%	2
Acanthuridae	*Acanthurus guttatus*	LC	20%	33%	1
Acanthuridae	*Acanthurus japonicus*	LC	38%	58%	1
Acanthuridae	*Acanthurus leucocheilus*	LC	32%	50%	1
Acanthuridae	*Acanthurus leucopareius*	LC	7%	19%	1
Acanthuridae	*Acanthurus leucosternon*	LC	24%	45%	1
Acanthuridae	*Acanthurus lineatus*	LC	20%	34%	1
Acanthuridae	*Acanthurus maculiceps*	LC	29%	45%	2
Acanthuridae	*Acanthurus mata*	LC	19%	33%	1
Acanthuridae	*Acanthurus monroviae*	LC	no data	no data	3
Acanthuridae	*Acanthurus nigricans*	LC	20%	33%	2
Acanthuridae	*Acanthurus nigricauda*	LC	20%	35%	3
Acanthuridae	*Acanthurus nigrofuscus*	LC	19%	32%	2
Acanthuridae	*Acanthurus nigroris*	LC	2%	6%	3
Acanthuridae	*Acanthurus nigros*	LC	4%	12%	3
Acanthuridae	*Acanthurus nubilus*	LC	28%	44%	1
Acanthuridae	*Acanthurus olivaceus*	LC	20%	33%	2
Acanthuridae	*Acanthurus polyzona*	DD	9%	33%	3
Acanthuridae	*Acanthurus pyroferus*	LC	20%	33%	1
Acanthuridae	*Acanthurus reversus*	LC	3%	5%	2
Acanthuridae	*Acanthurus sohal*	LC	18%	24%	1
Acanthuridae	*Acanthurus tennentii*	LC	24%	45%	2
Acanthuridae	*Acanthurus thompsoni*	LC	21%	35%	1
Acanthuridae	*Acanthurus triostegus*	LC	20%	35%	2

Acanthuridae	*Acanthurus tristis*	LC	29%	49%	3
Acanthuridae	*Acanthurus xanthopterus*	LC	21%	35%	3
Acanthuridae	*Ctenochaetus binotatus*	LC	21%	35%	2
Acanthuridae	*Ctenochaetus cyanocheilus*	LC	21%	35%	2
Acanthuridae	*Ctenochaetus flavicauda*	LC	3%	5%	1
Acanthuridae	*Ctenochaetus hawaiiensis*	LC	7%	13%	3
Acanthuridae	*Ctenochaetus marginatus*	LC	6%	14%	3
Acanthuridae	*Ctenochaetus striatus*	LC	20%	34%	2
Acanthuridae	*Ctenochaetus strigosus*	LC	2%	6%	3
Acanthuridae	*Ctenochaetus tominiensis*	LC	23%	38%	1
Acanthuridae	*Ctenochaetus truncatus*	LC	24%	44%	2
Acanthuridae	*Naso annulatus*	LC	18%	31%	1
Acanthuridae	*Naso brachycentron*	LC	20%	35%	1
Acanthuridae	*Naso brevirostris*	LC	19%	32%	2
Acanthuridae	*Naso caeruleacauda*	LC	29%	44%	1
Acanthuridae	*Naso caesius*	LC	5%	15%	1
Acanthuridae	*Naso elegans*	LC	19%	35%	1
Acanthuridae	*Naso fageni*	LC	33%	53%	3
Acanthuridae	*Naso hexacanthus*	LC	19%	33%	1
Acanthuridae	*Naso lituratus*	LC	20%	33%	2
Acanthuridae	*Naso lopezi*	LC	22%	36%	1
Acanthuridae	*Naso maculatus*	LC	9%	26%	2
Acanthuridae	*Naso mcdadei*	LC	26%	40%	3
Acanthuridae	*Naso minor*	LC	25%	40%	1
Acanthuridae	*Naso reticulatus*	DD	36%	56%	2
Acanthuridae	*Naso thynnoides*	LC	24%	39%	1
Acanthuridae	*Naso tonganus*	LC	20%	34%	1
Acanthuridae	*Naso tuberosus*	DD	12%	35%	1
Acanthuridae	*Naso unicornis*	LC	19%	32%	2
Acanthuridae	*Naso vlamingii*	LC	20%	35%	1
Acanthuridae	*Paracanthurus hepatus*	LC	21%	35%	1
Acanthuridae	*Prionurus biafraensis*	LC	no data	no data	3
Acanthuridae	*Prionurus chrysurus*	DD	40%	60%	3
Acanthuridae	*Prionurus laticlavius*	LC	13%	50%	3
Acanthuridae	*Prionurus maculatus*	LC	4%	18%	2
Acanthuridae	*Prionurus microlepidotus*	LC	3%	7%	3
Acanthuridae	*Prionurus punctatus*	LC	13%	46%	2
Acanthuridae	*Prionurus scalprum*	DD	40%	60%	3
Acanthuridae	*Zebrasoma desjardinii*	LC	18%	34%	1
Acanthuridae	*Zebrasoma flavescens*	LC	16%	27%	2
Acanthuridae	*Zebrasoma gemmatum*	DD	10%	33%	1
Acanthuridae	*Zebrasoma rostratum*	DD	3%	6%	2
Acanthuridae	*Zebrasoma scopas*	LC	20%	34%	2
Acanthuridae	*Zebrasoma veliferum*	LC	19%	32%	2

Acanthuridae	*Zebrasoma xanthurum*	LC	20%	31%	2
Scarinae	*Bolbometopon muricatum*	VU	19%	32%	1
Scarinae	*Calotomus carolinus*	LC	18%	32%	3
Scarinae	*Calotomus japonicus*	LC	20%	42%	3
Scarinae	*Calotomus spinidens*	LC	22%	37%	3
Scarinae	*Calotomus viridescens*	LC	14%	26%	3
Scarinae	*Calotomus zonarchus*	LC	2%	6%	3
Scarinae	*Cetoscarus bicolor*	LC	4%	8%	1
Scarinae	*Cetoscarus ocellatus*	LC	19%	32%	1
Scarinae	*Chlorurus atrilunula*	LC	13%	36%	1
Scarinae	*Chlorurus bleekeri*	LC	20%	33%	1
Scarinae	*Chlorurus bowersi*	NT	37%	57%	1
Scarinae	*Chlorurus capistratoides*	LC	31%	51%	1
Scarinae	*Chlorurus cyanescens*	LC	12%	35%	1
Scarinae	*Chlorurus enneacanthus*	LC	22%	42%	1
Scarinae	*Chlorurus frontalis*	LC	4%	12%	1
Scarinae	*Chlorurus genazonatus*	LC	4%	8%	1
Scarinae	*Chlorurus gibbus*	LC	4%	8%	1
Scarinae	*Chlorurus japanensis*	LC	22%	36%	1
Scarinae	*Chlorurus microrhinos*	LC	19%	31%	1
Scarinae	*Chlorurus oedema*	LC	37%	56%	1
Scarinae	*Chlorurus perspicillatus*	LC	2%	6%	1
Scarinae	*Chlorurus rhakoura*	LC	26%	43%	1
Scarinae	*Chlorurus sordidus*	LC	22%	38%	1
Scarinae	*Chlorurus spilurus*	LC	20%	33%	1
Scarinae	*Chlorurus strongylocephalus*	LC	24%	45%	1
Scarinae	*Chlorurus troschelii*	LC	40%	59%	1
Scarinae	*Cryptotomus roseus*	LC	14%	37%	3
Scarinae	*Hipposcarus harid*	LC	18%	33%	1
Scarinae	*Hipposcarus longiceps*	LC	19%	32%	1
Scarinae	*Leptoscarus vaigiensis*	LC	20%	34%	3
Scarinae	*Nicholsina denticulata*	LC	13%	49%	3
Scarinae	*Nicholsina collettei*	LC	no data	no data	3
Scarinae	*Nicholsina usta*	LC	15%	40%	3
Scarinae	*Scarus altipinnis*	LC	4%	12%	3
Scarinae	*Scarus arabicus*	LC	69%	84%	3
Scarinae	*Scarus caudofasciatus*	LC	19%	40%	1
Scarinae	*Scarus chameleon*	LC	19%	33%	1
Scarinae	*Scarus chinensis*	DD	20%	42%	no data
Scarinae	*Scarus coelestinus*	DD	14%	37%	3
Scarinae	*Scarus coeruleus*	LC	14%	37%	2
Scarinae	*Scarus collana*	LC	4%	8%	3
Scarinae	*Scarus compressus*	LC	13%	49%	3
Scarinae	*Scarus dimidiatus*	LC	21%	35%	2

Scarinae	*Scarus dubius*	LC	2%	6%	1
Scarinae	*Scarus falcipinnis*	LC	17%	39%	1
Scarinae	*Scarus ferrugineus*	LC	17%	23%	2
Scarinae	*Scarus festivus*	LC	24%	40%	1
Scarinae	*Scarus flavipectoralis*	LC	21%	34%	1
Scarinae	*Scarus forsteni*	LC	18%	31%	1
Scarinae	*Scarus frenatus*	LC	19%	33%	1
Scarinae	*Scarus fuscocaudalis*	LC	34%	51%	1
Scarinae	*Scarus fuscopurpureus*	LC	17%	23%	3
Scarinae	*Scarus ghobban*	LC	18%	34%	2
Scarinae	*Scarus globiceps*	LC	18%	31%	2
Scarinae	*Scarus guacamaia*	DD	14%	37%	3
Scarinae	*Scarus hoefleri*	LC	no data	no data	3
Scarinae	*Scarus hypselopterus*	NT	39%	59%	2
Scarinae	*Scarus iseri*	LC	14%	37%	3
Scarinae	*Scarus koputea*	LC	3%	5%	3
Scarinae	*Scarus longipinnis*	LC	3%	9%	1
Scarinae	*Scarus maculipinna*	DD	40%	60%	1
Scarinae	*Scarus niger*	LC	19%	33%	1
Scarinae	*Scarus obishime*	DD	20%	42%	3
Scarinae	*Scarus oviceps*	LC	18%	31%	1
Scarinae	*Scarus ovifrons*	DD	23%	45%	3
Scarinae	*Scarus perrico*	LC	13%	49%	3
Scarinae	*Scarus persicus*	LC	70%	85%	3
Scarinae	*Scarus prasiognathos*	LC	34%	53%	1
Scarinae	*Scarus psittacus*	LC	19%	33%	1
Scarinae	*Scarus pyrrostethus*	LC	19%	33%	3
Scarinae	*Scarus quoyi*	LC	30%	48%	2
Scarinae	*Scarus rivulatus*	LC	21%	35%	3
Scarinae	*Scarus rubroviolaceus*	LC	20%	34%	3
Scarinae	*Scarus russelii*	LC	20%	42%	1
Scarinae	*Scarus scaber*	LC	16%	32%	1
Scarinae	*Scarus schlegeli*	LC	18%	30%	3
Scarinae	*Scarus spinus*	LC	18%	31%	1
Scarinae	*Scarus taeniopterus*	LC	14%	37%	3
Scarinae	*Scarus tricolor*	LC	28%	46%	1
Scarinae	*Scarus trispinosus*	EN	13%	53%	3
Scarinae	*Scarus vetula*	LC	14%	37%	2
Scarinae	*Scarus viridifucatus*	LC	28%	48%	2
Scarinae	*Scarus xanthopleura*	LC	29%	46%	1
Scarinae	*Scarus zelindae*	DD	13%	53%	3
Scarinae	*Scarus zufar*	DD	70%	85%	3
Scarinae	*Sparisoma amplum*	LC	13%	53%	3
Scarinae	*Sparisoma atomarium*	LC	14%	37%	3

Scarinae	*Sparisoma aurofrenatum*	LC	14%	37%	3
Scarinae	*Sparisoma axillare*	DD	13%	53%	3
Scarinae	*Sparisoma chrysopterum*	LC	14%	37%	3
Scarinae	*Sparisoma cretense*	LC	no data	no data	3
Scarinae	*Sparisoma frondosum*	DD	13%	49%	3
Scarinae	*Sparisoma griseorubrum*	DD	13%	53%	3
Scarinae	*Sparisoma radians*	LC	14%	37%	3
Scarinae	*Sparisoma rubripinne*	LC	14%	37%	3
Scarinae	*Sparisoma strigatum*	LC	no data	no data	3
Scarinae	*Sparisoma tuiupiranga*	LC	13%	53%	3
Scarinae	*Sparisoma viride*	LC	14%	37%	3

Exclsively coral reef dependent	1
Primarily found in coral reef	2
coral reef [Mixed habitat	3
