# Supplementary material for: The Likelihood of Extinction of Iconic and Dominant Herbivores and Detritivores of Coral Reefs: The Parrotfishes and Surgeonfishes
Source: PLoS One. 2012 Jul 11;7(7):e39825. doi: 10.1371/journal.pone.0039825 (PMC3394754; doi:10.1371/journal.pone.0039825)
Supplement: Table S3 — Major threats identified for each species of parrotfish and surgeonfish. (PDF) [file pone.0039825.s003.pdf]

Scientific name	Habitat Loss	Fisheries	No major threats	Pollution/ Sedimentation	By-Catch	Unknown threats
Acanthurus achilles		1				
Acanthurus albipectoralis			1			
Acanthurus auranticavus			1			
Acanthurus bahianus		1				
Acanthurus bariene		1				
Acanthurus blochii		1				
Acanthurus chirurgus		1				
Acanthurus chronixis		1				
Acanthurus coeruleus		1				
Acanthurus dussumieri		1				
Acanthurus fowleri		1				
Acanthurus gahhm		1				
Acanthurus grammoptilus			1			
Acanthurus guttatus			1			
Acanthurus japonicus			1			
Acanthurus leucocheilus			1			
Acanthurus leucopareius			1			
Acanthurus leucosternon		1				
Acanthurus lineatus		1				
Acanthurus maculiceps			1			
Acanthurus mata		1				
Acanthurus monroviae			1			
Acanthurus nigricans			1			
Acanthurus nigricauda		1				
Acanthurus nigrofuscus			1			
Acanthurus nigroris			1			
Acanthurus nigros			1			
Acanthurus nubilus			1			
Acanthurus olivaceus			1			
Acanthurus polyzona						1
Acanthurus pyroferus			1			
Acanthurus reversus			1			
Acanthurus sohal		1				
Acanthurus tennentii		1				
Acanthurus thompsoni		1				
Acanthurus triostegus		1				
Acanthurus tristis			1			
Bolbometopon muricatum		1				
Calotomus carolinus	1					
Calotomus japonicus			1			
Calotomus spinidens	1					

Calotomus viridescens			1	
Calotomus zonarchus			1	
Cetoscarus bicolor			1	
Cetoscarus ocellatus	1			
Chlorurus atrilunula			1	
Chlorurus bleekeri	1	1		
Chlorurus bowersi		1		
Chlorurus capistratoides			1	
Chlorurus cyanescens	1			1
Chlorurus enneacanthus			1	
Chlorurus frontalis		1		
Chlorurus genazonatus			1	
Chlorurus gibbus			1	
Chlorurus japanensis	1	1		1
Chlorurus microrhinos	1	1		
Chlorurus oedema			1	
Chlorurus perspicillatus		1		
Chlorurus rhakoura			1	
Chlorurus sordidus			1	
Chlorurus spilurus		1		
Chlorurus strongylocephalus		1		
Chlorurus troschelii			1	
Cryptotomus roseus			1	
Ctenochaetus binotatus			1	
Ctenochaetus cyanocheilus			1	
Ctenochaetus flavicauda			1	
Ctenochaetus hawaiiensis		1		
Ctenochaetus marginatus			1	
Ctenochaetus striatus		1		
Ctenochaetus strigosus			1	
Ctenochaetus tominiensis			1	
Ctenochaetus truncatus			1	
Hipposcarus harid		1		
Hipposcarus longiceps				
Leptoscarus vaigiensis		1		1
Naso annulatus			1	
Naso brachycentron			1	
Naso brevirostris			1	
Naso caeruleacauda			1	
Naso caesius			1	
Naso elegans			1	
Naso fageni			1	
Naso hexacanthus		1		
Naso lituratus		1		1

Naso lopezi		1				
Naso maculatus		1				
Naso mcdadei						1
Naso minor			1			
Naso reticulatus						1
Naso thynnoides		1				
Naso tonganus			1			
Naso tuberosus						1
Naso unicornis		1				
Naso vlamingii		1				
Nicholsina collettei			1			
Nicholsina usta			1			
Paracanthurus hepatus	1	1				
Prionurus biafraensis			1			
Prionurus chrysurus						1
Prionurus maculatus			1			
Prionurus microlepidotus			1			
Prionurus scalprum			1			
Scarus altipinnis		1				
Scarus arabicus			1			
Scarus caudofasciatus			1			
Scarus chameleon		1				
Scarus chinensis						1
Scarus coelestinus		1				
Scarus coeruleus		1				
Scarus collana			1			
Scarus dimidiatus		1		1		
Scarus dubius			1			
Scarus falcipinnis			1			
Scarus ferrugineus			1			
Scarus festivus			1			
Scarus flavipectoralis		1				
Scarus forsteni		1				
Scarus frenatus		1				
Scarus fuscocaudalis			1			
Scarus fuscopurpureus			1			
Scarus ghobban		1			1	
Scarus globiceps		1				
Scarus guacamaia	1	1				
Scarus hoefleri			1			
Scarus hypselopterus		1				
Scarus iseri			1			
Scarus koputea			1			
Scarus longipinnis			1			

Scarus maculipinna						1
Scarus niger		1				
Scarus obishime						
Scarus oviceps			1			
Scarus ovifrons		1		1		
Scarus persicus			1			
Scarus prasiognathos			1			
Scarus psittacus		1				
Scarus pyrrostethus		1			1	
Scarus quoyi						
Scarus rivulatus		1				
Scarus rubroviolaceus		1				
Scarus russelii			1			
Scarus scaber			1			
Scarus schlegeli		1				
Scarus spinus		1				
Scarus taeniopterus			1			
Scarus tricolor		1				
Scarus trispinosus		1				
Scarus vetula		1				
Scarus viridifucatus			1			
Scarus xanthopleura			1			
Scarus zelindae		1				
Scarus zufar						1
Sparisoma amplum		1				
Sparisoma atomarium			1			
Sparisoma aurofrenatum			1			
Sparisoma axillare		1				
Sparisoma chrysopterum		1				
Sparisoma cretense			1			
Sparisoma frondosum		1				
Sparisoma griseorubrum						1
Sparisoma radians			1			
Sparisoma rubripinne		1				
Sparisoma strigatum		1	1			
Sparisoma tuiupiranga			1			
Sparisoma viride		1				
Zebrasoma desjardinii			1			
Zebrasoma flavescens		1				
Zebrasoma gemmatum						1
Zebrasoma rostratum						1
Zebrasoma scopas		1				
Zebrasoma veliferum	1	1				
Zebrasoma xanthurum			1			
